# Integrative analysis of proteomic and metabonomics data for identification of pathways related to *Rhizoma Paridis*-induced hepatotoxicity

**DOI:** 10.1038/s41598-020-63632-1

**Published:** 2020-04-16

**Authors:** Chongjun Zhao, Mingshuang Wang, Jianmei Huang, Zhe Jia, Xia Zhao, Erwen Li, Ziying Wei, Ying Dong, Wenxue Liu, Ting Han, Ying Liu, Farong Li, Ruichao Lin

**Affiliations:** 1 Beijing Key Laboratory for Quality Evaluation of Chinese Materia Medica, School of Chinese Materia Medica, Beijing University of Chinese Medicine, Beijing, P.R. China; 2 Key Laboratory of Ministry of Education for Medicinal Resources and Natural Pharmaceutical Chemistry, National Engineering Laboratory for Resource Developing of Endangered Chinese Crude Drugs in Northwest of China, Shanxi Normal University, Xi’an, China; 3 Key Laboratory of Tropical Translational Medicine of Ministry of Education, Hainan Key Laboratory for Research and Development of Tropical Herbs, School of Pharmacy, Hainan Medical University, Haikou, 571100 China

**Keywords:** Hepatotoxicity, Protein-protein interaction networks

## Abstract

Clinical reports on hepatotoxicity that arise from *Rhizoma Paridis* have recently received widespread attention. Because the hepatotoxicity mechanism is little understood, this research strived to investigate the hepatotoxicity mechanism of *Rhizoma Paridis* extracts based on iTRAQ quantitative proteomics and metabonomics. The extraction solutions were administrated to rats for 7 days by gavage, and the hepatotoxicity was assessed through quantification of biochemical indexes and Oil red O staining. Additionally, the mechanism of hepatotoxicity was investigated by metabonomics based upon GC-MS and iTRAQ quantitative proteomics. The biochemical and histopathological analysis stood out that *Rhizoma Paridis* extract could induce liver injury, which was proved by the formation of fat droplets, the changes of mitochondrial structure, and biochemical parameters. The iTRAQ proteomics and metabonomics revealed that *Rhizoma Paridis-*induced hepatotoxicity was chiefly connected with the abnormal activity of mitochondrion function, which brought about oxidative stress injuries and inflammation, finally causing cell apoptosis. Collectively, we have provided previously uncharacterized hepatotoxic mechanism induced by *Rhizoma Paridis* and a reference to ensure its safe use in the future.

## Introduction

*Rhizoma Paridis*, the roots of the *Paris polyphylla* Smith var. (Franch.), was first recorded in ‘Shennong Herb’ and Li Shizhen’s ‘Compendium of Materia’. The prepared *Rhizoma Paridis has* remarkable therapeutic effects on fractures, parotitis, hemostasis, snake bite, and abscess in the clinic for thousands of years. In addition, modern pharmacology indicated that Raw *Rhizoma Paridis* has beneficial impact on anti-tumor, immunity adjustment, analgesia, and anti-inflammation^1–3^. However, clinical researches regarding adverse reactions caused by *Rhizoma Paridis* and its preparations^4^, especially hepatotoxicity, have attracted significant attention in recent years^5^. The Chinese Pharmacopoeia clearly describes its hypotoxicity and reminds the patient and doctor to note the possible problems of orally ingesting *Rhizoma Paridis* and its drug preparations in high doses or over prolonged periods and when taken with other liver-damaging drugs. Despite these warnings, the mechanisms remained little known^6^.

Recently, omic technologies are becoming important *in vitro* and *in vivo* tools for identifying potential hepatotoxicity mechanisms. The proteomics can identify the differentially expressed proteins that occur in a specific function, which may involve disease- or disorder-related changes in transcription, translation, transport, degradation, and covalent modification^7,8^. Metabolomics is another powerful technology for identifying a number of low-molecular-weight endogenous metabolites and assessing the dynamic variations in the biological samples in response to different stimuli^9,10^. These provide a lot of biological information and allow in-depth analysis of the biochemical and elementary mechanisms in alternative models consequently as to formulate the beneficial strategies to control adverse effects.

Here, to clarify *Rhizoma Paridis-*induced hepatotoxicity and the mechanism, metabolites and protein profiles derangement of liver tissues collected from experimental rats treated by *Rhizoma Paridis* were analyzed. In accordance with the result of metabolomics, iTRAQ quantitative proteomics, the liver histopathology, the biochemical index, we found that *Rhizoma Paridis-*induced hepatotoxicity can be chiefly attributed to the imbalance of energy and lipid metabolism, so that mediated a procession of pathological responses including inflammation, oxidative stress injury, and apoptosis, ultimately engendering liver injury. Collectively, our data provided cognizance of the rational clinical application of *Rhizoma Paridis* and supported a promising strategy for detoxifying.

## Materials and Methods

All animals were utilized in this study referring to the Animal Ethics Committee of Beijing University of Traditional Chinese Medicine. Guiding by the Animal Management Rules of the Ministry of Science and Technology of the People’s Republic of China, all animals recieved care and raising with standard food and water. The Spaifu Biotechnology Co. LTD (Beijing, China) provided us with eighty male Sprague-Dawley rats (200 ± 10 g), whose license number was 11401500039719. We controlled the experimental condition, which animals kept in, that the temperature is 23 °C ± 2 °C; relative humidity is 60% ± 5% and the light/dark cycle is 12 h alternating. Distilled water was used as the vehicle control. As for the treatment groups, the animals, randomly divided into 3 groups of 12 rats, were administrated with 0.0387 g/100 g (L), 0.1161 g/100 g (M) and 0.3483 g/100 g (H) Rhizoma Paridis by gavage, respectively. All the rats need one week of acclimation before experiment.

After 7 days, all mice were euthanized following animal ethical standards. The small section of hepatic tissue was kept in 4% paraformaldehyde solution or 2.5% glutaraldehyde for histological evaluation. Liquid nitrogen was used to freeze the rest of the hepatic samples in a snap for some determination of relative genes, protein expression, and physiological parameters.

### Histopathology and clinical biochemistry

4% paraformaldehyde solution were used for fixing the small section of hepatic tissue for histological evaluations. And paraffin embedded the fixed hepatic samples so that they were cut into 5-µm thickness. Hematoxylin and eosin (H&E) and Oil Red O staining were performed in hepatic sections to visulize the necrotic areas under the microscope (Olympus DX45, Tokyo, Japan).

Guiding by the manufacturer’s instructions(Nanjing Jiancheng Bioengineering Institute, Nanjing, China), the biochemistry parameters detection were carried out to pinpoint molecular changes associated with liver injury, including malondialdehyde (MDA), super oxide dismutase (SOD), ATP concentration, succinate dehydrogenase (SDH), and nicotinamide adenine dinucleotide oxidase (NOX). The sections(5 μm), stained with hematoxylin and eosin, were produced by the tissue slicer (Leica RM2016, Shanghai, China). The microscope (Olympus DX45, Tokyo, Japan) was used for undergoing histological observation.

### iTRAQ-based quantitative proteomic analysis

According to the previously described, quantitative proteomic analysis protocol was provided in the supporting information with modification details. In brief, proteins were extracted from tissue samples using triethylammonium formate (TEAB)/acetone. One replicate was made of three individuals of H group (n = 3). The BCA kit (Nanjing Jiancheng Bioengineering Institute, China) was carried out to measure concentrations of protein in extracts. Whereafter, TEAB was preformed to reduce and alkylate protein(100 μg) for each sample. With that, trypsin acted on each sample, which subsequently was labeled by iTRAQ reagents (TMT Reagent-Multiplex Buffer Kit; ABSCIEX, Darmstadt, Germany). Buffer solution (100 μL, 10 mM ammonium formate, Ph=10) re-dissolved the dried and pooled peptides, which was followed by samples separation (50 μL) employing high-pH reverse-phase HPLC (Thermo Scientific). In doing so, 10 peptides were isolated from the different samples and then fractionated samples were centrifuged, condensed, and dried in sequence. The 10 μL of buffer (1%ACN, 0.1% FA) were used for re-dissolving fractionated samples, and 5 μL of the prepared sample was analyzed by LC-MS, which was performed by the online Nano-RPLC HPLC (Easy-nLC 1000 system) with the Orbitrap Fusion system (Thermo Fisher Scientific, USA). The peptides were separated on a C18 reversed-phase column (PepMap100, C18 2 μm 75 μm × 250 mm NanoViper, Thermo Fisher Scientific). The differentially expression proteins were stood out with p value < 0.05 and a fold difference > 1.2 in term of abundance level during experimental group in comparison to control.

To ensure accuracy of result, an extensive series of quality control (QC)/assurance procedures was employed, including the enzymatic efficiency, tagging efficiency, the coverage of the protein, unique peptide number and cumulation percentage, and mass error distribution.

### GC-MS-based metabolomic analysis

Nine samples from the H group were involved in metabolomic analysis. A time-of-flight mass spectrometry (GC-TOF/MS) system (Pegasus HT, Leco Corp., St, Joseph, MO, USA) was performed to identify metabolites in liver. The system was conducted with a Gersted multipurpose Sample mps2 with dual heads (Gerstel, Muehlheim, germang) and an Agilent 7890B gas chromatography. As previously described, the sample preparation procedures was executed^11,12^. Furthermore, to ensure consistently high quality of data, an extensive series of quality control (QC)/assurance procedures was employed. Detailed information regarding this experiment condition was listed in the Supplementary Table 1.

To visualize the intragroup variations, principal component analysis (PCA) was executed, which can detect potential outliers. The resulted matrix was also applied to PLS-DA and OPLS-DA analyses simultaneously to provide profile visualization and differentiation regarding the sets of data, respectively.

To screen reliable metabolite marker, volcano-plot was performed with variable reliability (correlation coefficients, Corr.) and contribution (variable importance in projection, VIP). The typical rule, a p < 0.05 and VIP > 1, was used as selecting relevant variables

### Integrative analysis

To achieve an additional layer of understanding about *Rhizoma Paridis-*induced hepatotoxicity, the proteomics data was integrated with metabolomics data using MetaboAnalyst 3.0 software as subsquent pathway analysis. Functional annotation of those differential datas were carried out by Gene Ontology (GO),and they were assigned to KEGG pathways. Functional enrichment analysis was used to insight into the means of bio-information annotation and analysis.

### Western blot and quantitative Real-Time PCR (qRT-PCR)

Western blot and qRT-PCR assay were carried out in triplicate according to a previous report^13^. The DNA damage inducible transcript 3 (CHOP), Immunoglobulin heavy chain-binding protein homolog (BIP), Caspase-3, interleukin 1 beta (IL-1β), cytochrome c oxidase subunit 4I1 (COXIV) (Abcam, Beijing, China) were procured from the Beijing Biodee Biotechnology Co. Ltd (Beijing, China).All primers applied in this experiment were listed in the Supplementary Table S2.

### Data analysis

XploreMET (v3.0, Metabo-Profile, Shanghai, China) is a powerful 1-STOP solution for GC-MS-based metabolomics through more than a decade of research and development. The software streamlines procedures for processing of the raw data, peak deconvulation, compound annotation, statistical examination, pathway analysis, and project report within minutes of completing the analytical sequence.

Using XploreMET processed the data obtained from GC-TOF/MS. Furthermore, the system can automatedly creat reference database according to the data from pooled QC samples and finish the metabolite signal alignment, missing value correction and imputation, and QC correction.

Comparable data vectors, transformed from each data set, were applied to statistical analysis. Mean-centered and scaled by the standard deviation was used for all measurements.

### Ethical approval

The experiment was in accordance with the Animal Management Rules of the Ministry of Science and Technology of the People’s Republic of China for experimental care and use of animals and approved by the Animal Ethics Committee of Beijing University of Traditional Chinese Medicine.

## Result

### Characterization of rhizoma paridis-triggered liver injury

**Figure 1:**
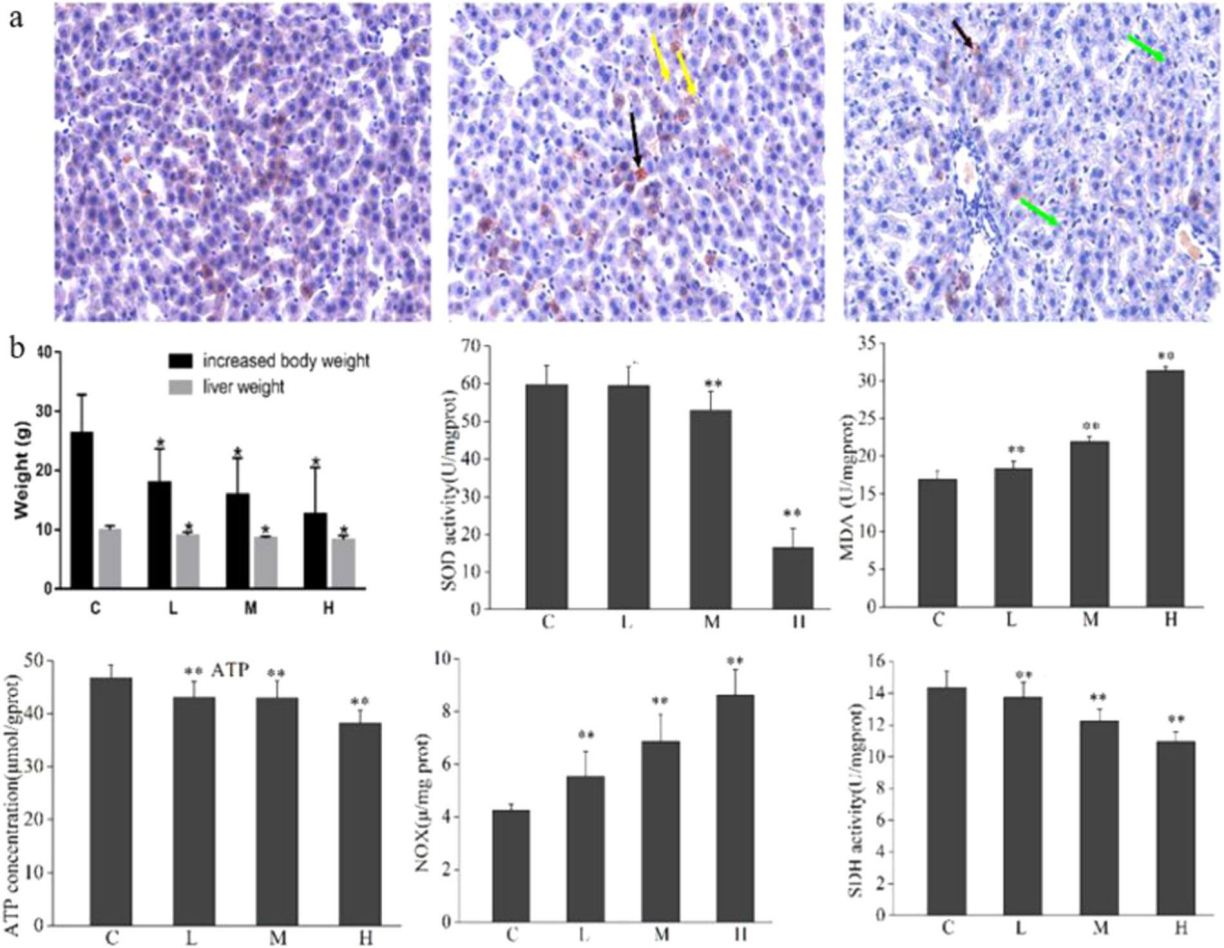
(**a**) Oil red O staining of livers from control and *Rhizoma Paridis*-treated rat. Fat droplets, vacuolation, hepatocyte necrotic foci are indicated as black, yellow, and blue arrows, respectively. (**b**) Tissue SOD, SDH, and NOX enzyme activity, ATP concentration, and MDA level from control and treated rat.

After a 7-day administration of *Rhizoma Paridis*, the overall toxicity of rats was observed in all groups. In the whole experiment, no death was found. Compared with the control group, body weight increase and liver weight decrease were significant, which meant that the *Rhizoma Paridis* caused liver toxicity in the rats to some extent. The histopathological examination with Oil red O showed that liver sections from the control rats showed normal liver histologic architecture. However, disordered organizational structure, fat droplets, and vacuolation were observed in the *Rhizoma Paridis*-treated livers (Fig. 1a). Furthermore, compared with the control group, the level of increased malondialdehyde (MDA) in the liver tissue of the treated group and decreased hepatic antioxidant enzyme activity, such as super oxide dismutase (SOD), were extremely significant (Fig. 1b). ATP concentration and succinate dehydrogenase (SDH) activity were significantly inhibited, whereas nicotinamide adenine dinucleotide oxidase (NOX) activity was elevated. According to these results, we speculated that *Rhizoma Paridis-*induced acute liver injury might be triggered by lipid and energy metabolism dysfunctions, the oxidative stress injury and ultimately resulted in cell apoptosis.

### Alteration of metabolites in liver

**Figure 2:**
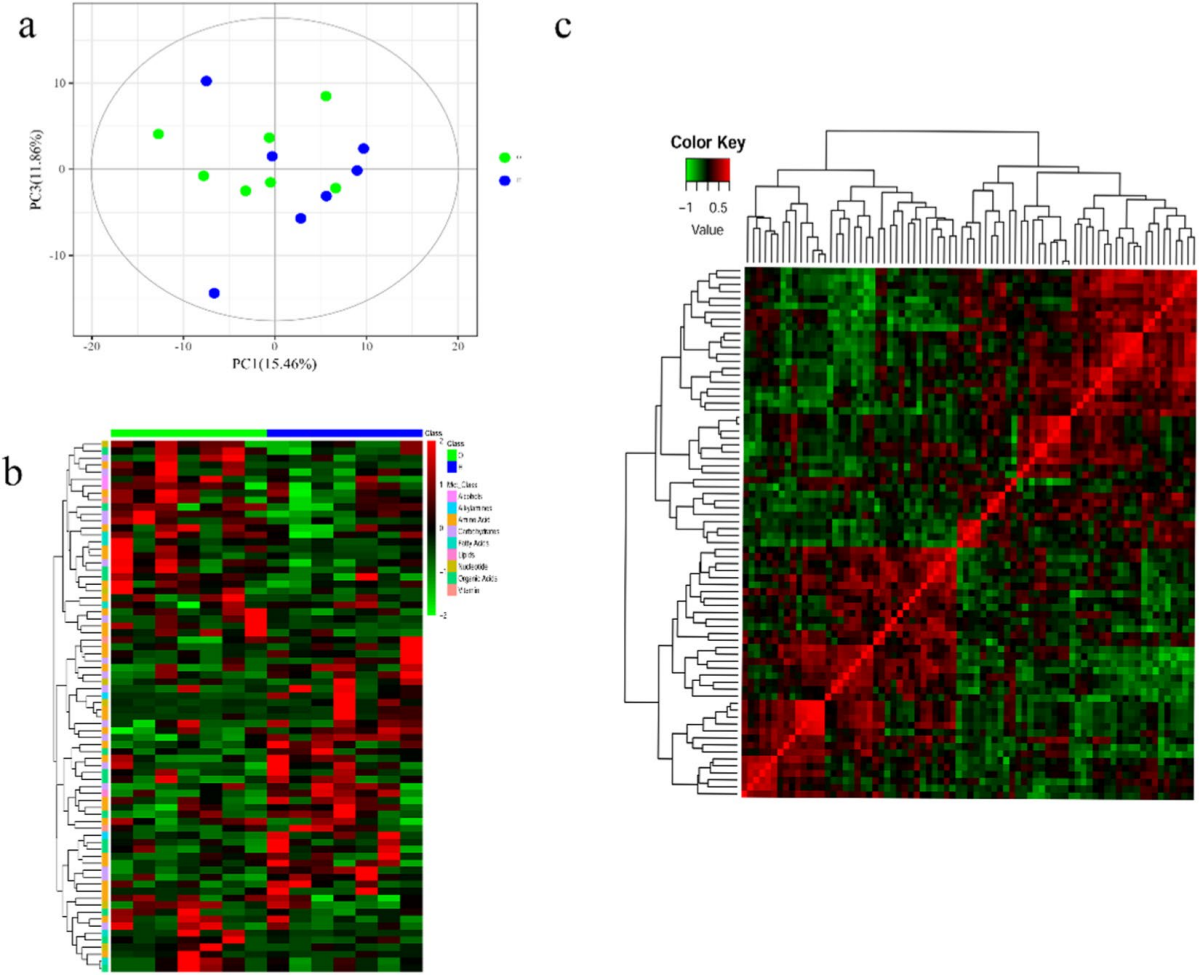
(**a**) PCA score scatter plots of the chromatograms results (n = 7, green circle dot) and high-dose treatment (n = 7, blue circle dot) of *Rhizoma Paridis*. (**b**) Pearson correlations of the metabolites, and the significant linear correlation tend to be grouped together. (**c**) Heatmap of z-score.

To analyze the effect of *Rhizoma Paridis* treatment on metabolic alterations, we assessed liver metabolites by GC-MS. To gain an overview of GC-MS data, we performed PCA across all samples and to detect outliers. In Fig. 2a, the result suggested overall profile similarities and dissimilarities among the groups. As illustrated in Fig. 2b, the cluster analysis of metabolites showed that the expression patterns of different metabolites in the two groups (including the up-regulation and down-regulation) were different. Also, the interrelationship of metabolites between the different groups was conducted using Pearson’s correlation and gained the Pearson’s correlation coefficient.(Fig. 2c).

**Figure 3:**
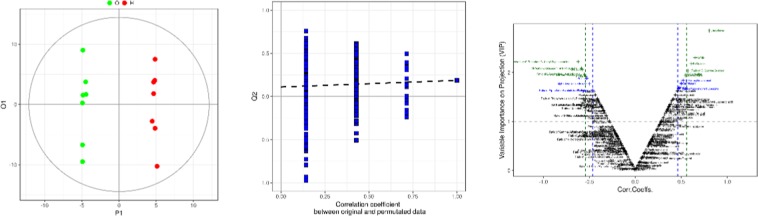
OPLS-DA score scatter plots obtained for metabolomic data corresponding to the control group (n = 7, green circle dot) and *Rhizoma Paridis* high-dose treatment group (n = 7, red circle dot); statistical validation of the respective OPLS-DA models by permutation testing (100 permutations); OPLS-DA volcano plot achieved for the respective pairwise comparison with the variables colored depending on their VIP value.

Supporting the result from PCA models, we achieved the visual result about differentiation among the groups by using PLS-DA and OPLS-DA analyses to avoid interference from unwanted variations in our metabonomics data set.(Fig. 3). The validity of the models constructed from the rat liver spectral data was confirmed by permutation tests and cross-validation parameter Q2. Then, the OPLS-DA score plots and corresponding loading plots based on the GC-MS liver tissue data for the pairwise groups show a clear separation between different treated groups and the corresponding controls. Furthermore, the metabolites for positively correlated subjects were also successfully displayed, suggesting that *Rhizoma Paridis* exposure obviously affected rat hepatic metabolomics.

**Figure 4:**
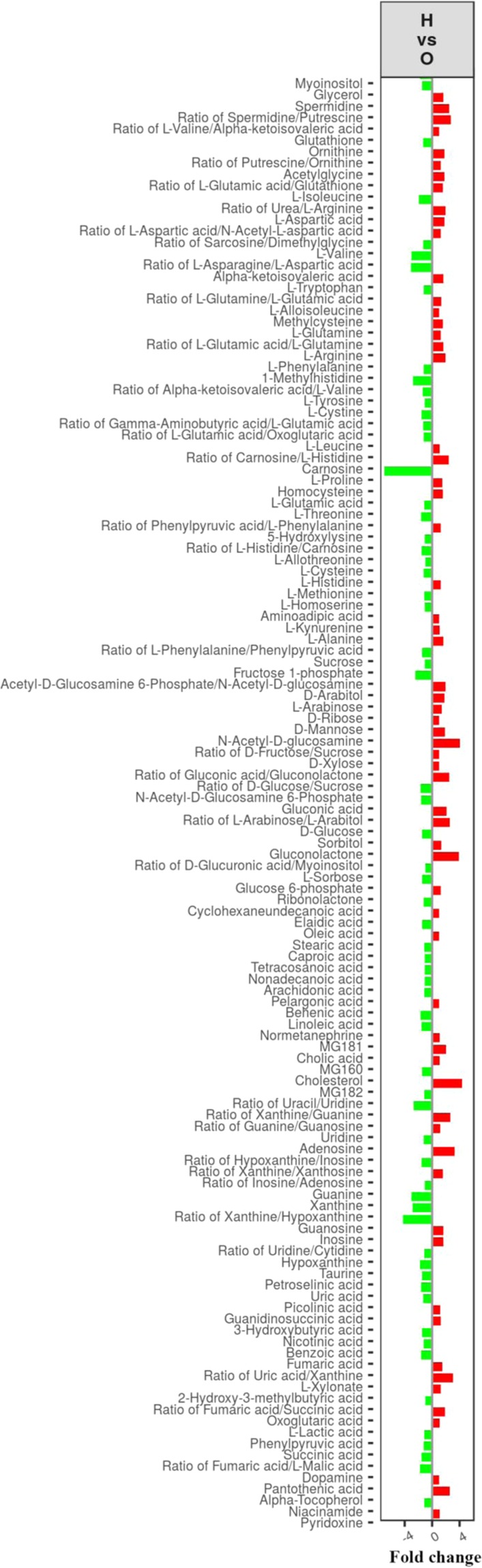
Differential metabolite profiles across groups between the control and treated group.

The extracted variables that contributed most to group distinction were screened as the potential biomarkers for *Rhizoma Paridis* administration. The significantly changed metabolites were chosen following the criteria using VIP ≥ 1 and Corr. Coeffs. at p < 0.05. Of all measured metabolites, the metabolites that were significantly altered and the change trend compared with the control were shown in the Fig. 4. The fold changes of the ratio 2 vs. 1.3 vs. 1, etc., were used to display the trend. These results suggested that *Rhizoma Paridis* exposure can significantly change the metabolites of carbohydrate metabolism, lipid and fatty acid metabolism, amino acid metabolism, and nucleotide metabolism.

Metabolic pathway enrichment analysis (MPEA) was performed by the untargeted metabolic profiling platform X plore MET (Metabo-Profile, Shanghai, China) to identify the most relevant metabolic pathways, which are shown in Table 1 (p-value <0.05). Notably, the target metabolites that were up-regulated or down-regulated in the treated groups were also listed.

**Table 1:** Key metabolic pathways and metabolism affected by treatment with *Rhizoma Paridis*.

PathwayName	P.hyper	Impact	Up	Down
Arginine biosynthesis	1.29e-06	0.56	L-Glutamic acid; L-Aspartic acid; Oxoglutaric acid; L-Arginine; L- Glutamine	Fumaric acid; Ornithine
D-Glutamine and D-glutamate metabolism	1.04e-03	0.67	L-Glutamic acid; Oxoglutaric acid; L-Glutamine	
Valine, leucine and isoleucine biosynthesis	5.24e-03	0.50	L-Isoleucine	Alpha-ketoisovaleric acid; L-Threonine
Histidine metabolism	6.08e-03	0.27	Carnosine; L-Glutamic acid; L-Aspartic acid	1-Methylhistidine
Galactose metabolism	8.39e-03	0.06	D-Glucose	Glycerol; Myoinositol; Sorbitol; Sucrose
Alanine, aspartate and glutamate metabolism	9.83e-03	0.48	L-Glutamic acid; L-Aspartic acid; Oxoglutaric acid; L-Glutamine	Fumaric acid
Nitrogen metabolism	3.10e-02	0.25	L-Glutamic acid; L-Glutamine	
Butanoate metabolism	3.32e-02	0.20	L-Glutamic acid; Oxoglutaric acid; 3-Hydroxybutyric acid	
Arginine and proline metabolism	3.43e-02	0.25	L-Glutamic acid; L-Arginine; Spermidine	L-Proline; Ornithine
Glutathione metabolism	4.40e-02	0.11	L-Glutamic acid; Spermidine	Ornithine; L-Cysteine
Taurine and hypotaurine metabolism	0.054	0.50		Taurine; L-Cysteine
Pantothenate and CoA biosynthesis	0.062	0.11	L-Aspartic acid	Alpha-ketoisovaleric acid; L-Cysteine
beta-Alanine metabolism	0.079	0.14	Carnosine; L-Aspartic acid; Spermidine	
Neomycin, kanamycin and gentamicin biosynthesis	0.095	1.00	D-Glucose	

### Expressions of Rhizoma Paridis-regulated proteins in liver

Totally, 3810 proteins were identified, and there were 3672 proteins quantified by using iTRAQ labeling. Among those quantified, 50 proteins were affected by high-dose treatment with *Rhizoma Paridis*.

To reduce the probability of false peptide identification, we selected peptides, with a fold change of > 1.2 or < 0.8, p-values < 0.05, as designate differentially expressed proteins in the analysis.

To elucidate the biological significance of these different modified proteins, gene ontology (GO) analysis was used to categorize these differential proteins according to their molecular function (MF), cellular component (CC), and biological process (BP).

**Figure 5:**
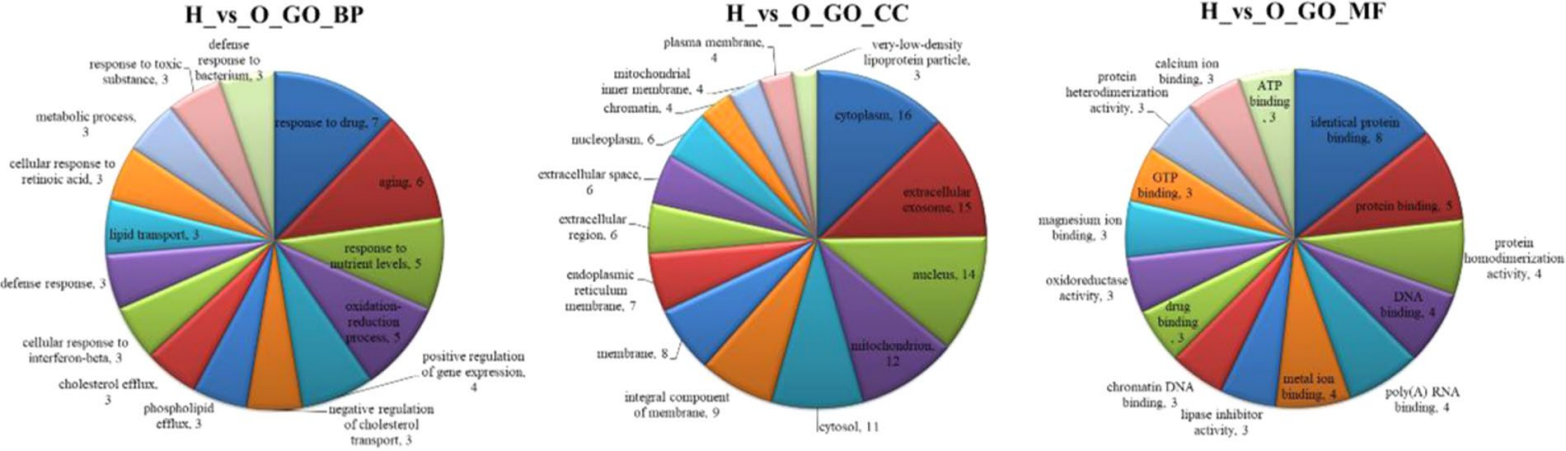
Classification of proteins based on their molecular function (MF), cellular component (CC), and biological process (BP) extracted from experimental rat liver.

To deeper analysis of differential proteins, we have divided them into various categories (Fig. 5). In accordance with the result of biological processes, regulated proteins were found to be involved in response to drugs, aging, response to nutrient level, or the oxidation-reduction process in the H group. The proteins affected in the H group covered extensive molecular functions, especially, binding. The categorization of cellular components showed that differentially expressed proteins were cheifly distributed in the cytoplasm, extracellular exosome, nucleus, mitochondria, and cytosol. Similar to previous evidences, the toxic effects of Rhizoma Paridis were related to lipid and energy metabolism dysfunction and that Rhizoma Paridis mediated oxidative stress and inflammation.

**Figure 6:**
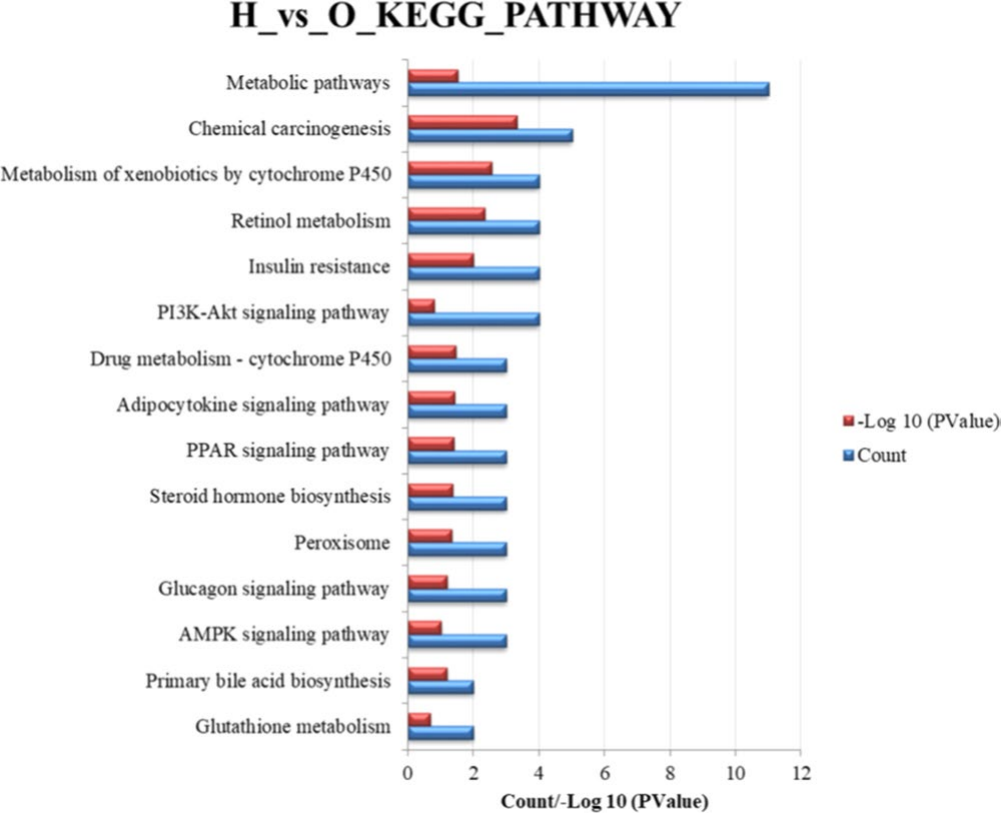
IPA analysis of differentially expressed proteins in the liver and the top 10 pathways affected in liver treated with *Rhizoma Paridis*.

Deeper analysis of the differential expressed proteins by using KEGG Pathway analysis, the top 10 pathways of each group are shown in Fig. 6. The major pathways in the treated group were relevant to the liver injury, including the metabolism pathway, steroid hormone biosynthesis, retinol metabolism, chemical carcinogenesis, arachidonic acid metabolism, and so on. Meanwhile, the metabolic pathways, retinol metabolism, insulin resistance, chemical carcinogenesis, PI33K-AKt signaling pathway and metabolism of xenobiotics by cytochrome P450 are the main pathways involving liver injury. Remarkably, there were six increased target proteins with 11 down-regulated proteins in our data, which might further highlight the specific to *Rhizoma Paridis* hepatotoxicity.

### Integrative proteomics and metabolomics analysis

Based on the dramatically changed proteins and metabolites, the Integrative Proteomics and Metabolomics Analysis were used for further holistic analysis to interpret a biological systems.

**Figure 7:**
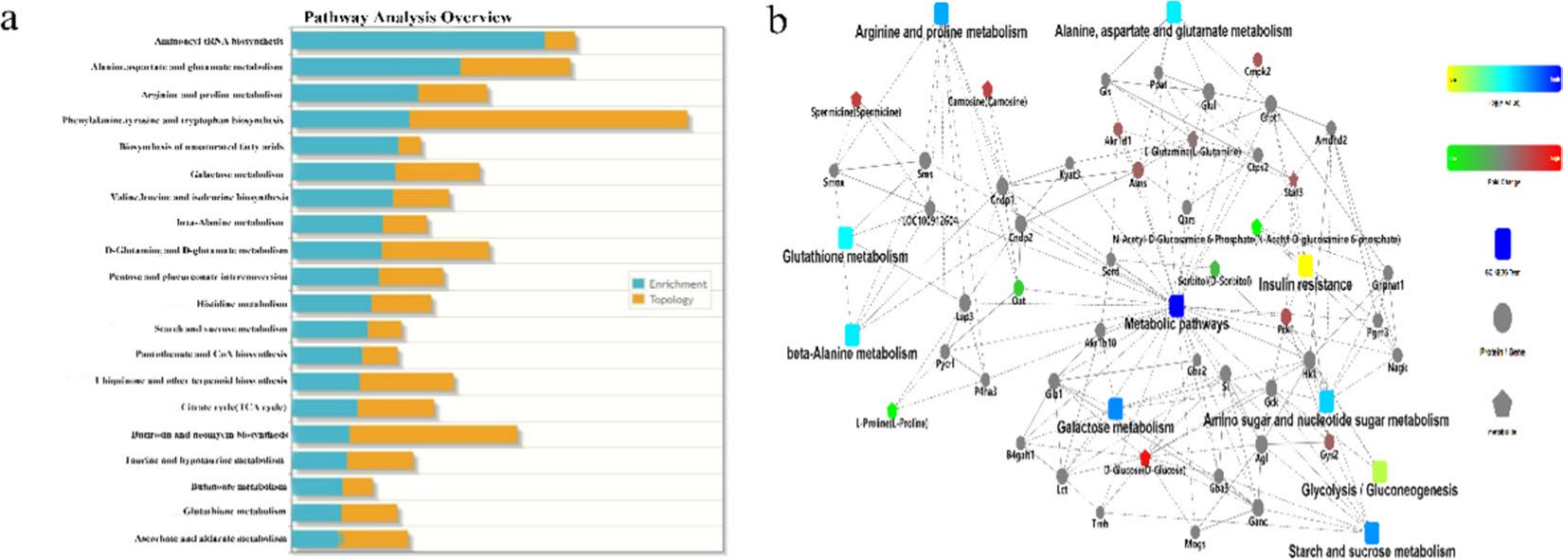
The Integrative Proteomics and Metabolomics Analysis interprets a further holistic biological systems. (**a**) Schematic of the Integrative analysis showing the visualization of the differentially abundant proteins and metabolites. (**b**) The differential proteins and metabolites were plotted for network. In doing this, we identified molecules inducing hepatotoxicity by *Rhizoma Paridis*.

First, we built a gene-metabolite functional enrichment in accordance with the data on differential proteins and metabolites obtained *in vivo* by carrying out MetaboAnalyst 3.0 software. In doing so, we found that both differential proteins and metabolites could be enriched in aminoacyl-tRNA biosynthesis; alanine, aspartate, and glutamate metabolism; arginine and proline metabolism; biosynthesis of unsaturated fatty acids; galactose metabolism; valine, leucine, and isoleucine biosynthesis; and so on (Fig. 7a). Furthermore, using IPA software, we identified the major signaling pathways in rat liver interfered with *Rhizoma Paridis*, which was visualized in network diagrams. In accordance with portion of the differential proteins and metabolites, we mapped the top-ranked network by IPA. Conditionally, the content was used to generate network with a score of ≥ 2 and a p-value <0.05 (Fig. 7).

### Verification of altered protein expressions

To identify potential early gene biomarkers of *Rhizoma Paridis* toxicity, real-time polymerase chain reaction (qRT-PCR) analysis and western blot were used to confirm the potential hepatotoxicity effect of *Rhizoma Paridis* on the associated biological processes, the related genes, and the proteins.

**Figure 8:**
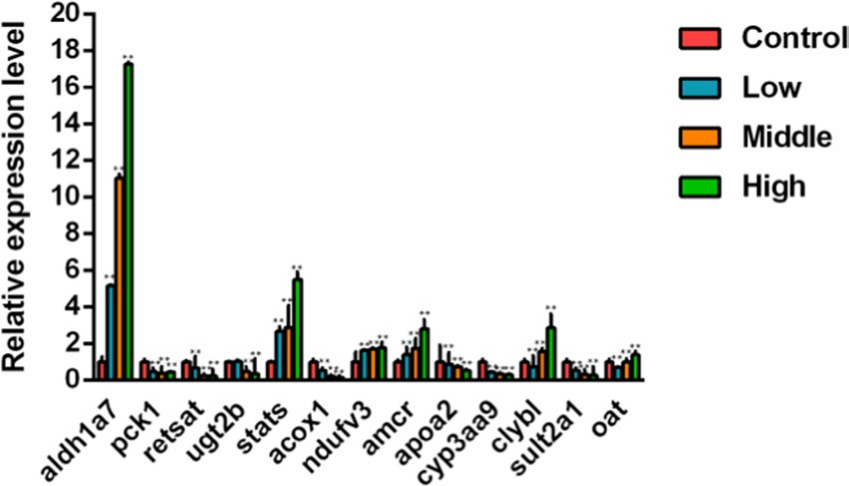
qRT-PCR expression ratios for selected genes expressed in the liver after 7 daily different doses of *Rhizoma Paridis*. Data were represented as a mean factor ± SEM in comparison with the control group. **p < 0.01.

Changes in transcription of genes that corresponded to identified proteins are shown in Fig. 8. For rat treated with high-dose Rhizoma Paridis, levels of mRNA for of aldh1a7, stats, ndufv3, amacr, clybl, and oat were greater compared with the control. At this dose of *Rhizoma Paridis*, levels of mRNA for pck1, retsat, ugt2b, acox, apoa2, cyp3a9, and sult2a1 were significantly less.

**Figure 9:**
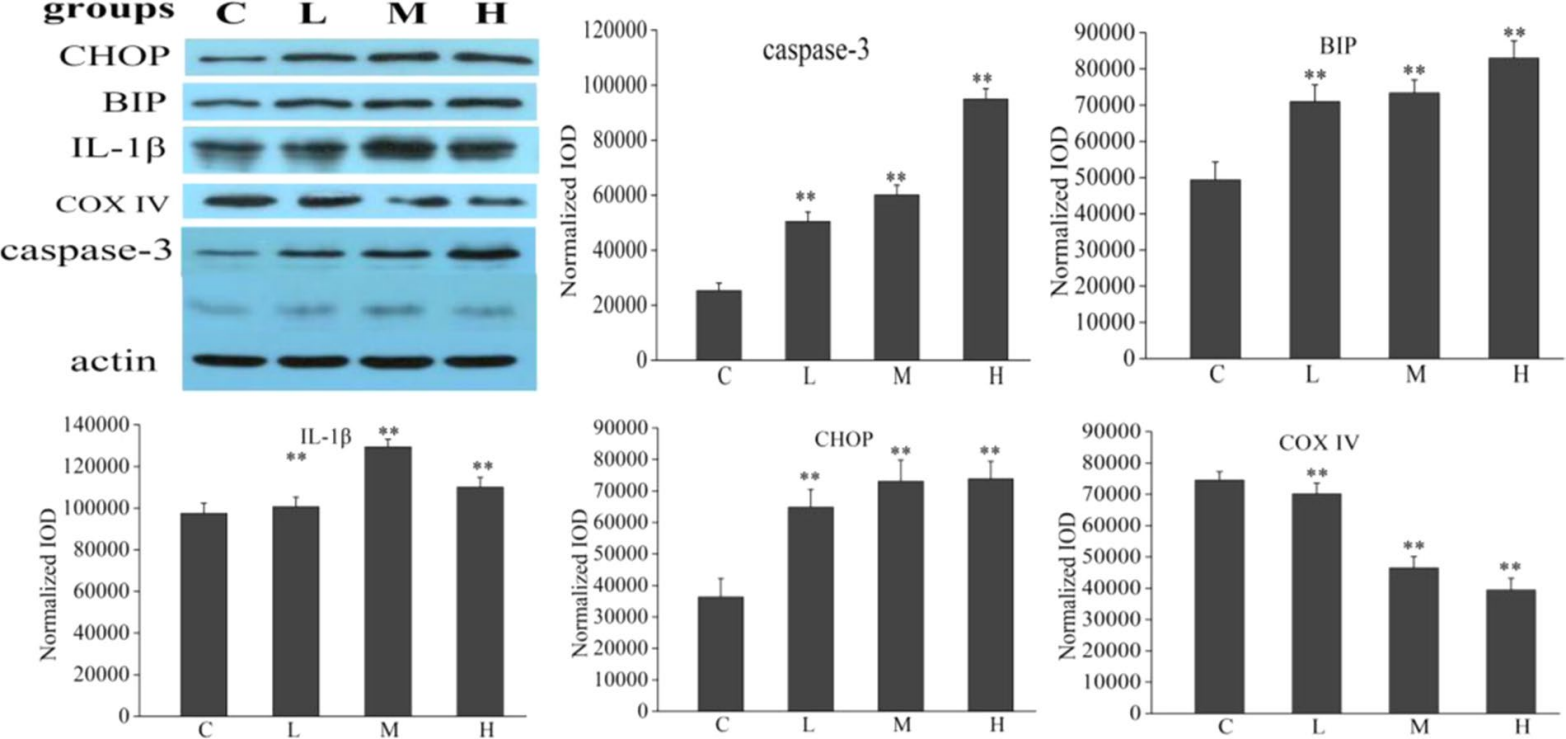
Western blotting analysis of CHOP, BIP, IL-1β, Caspase-3 COX IV concentrations in liver tissue from control and treated rat. The values are mean ± SEM. **p < 0.01.

Alterations of the proteins associated with apoptosis (caspase-3) and endoplasmic reticulum stress (BIP), lipid metabolism (CHOP), and inflammatory response (IL-1β) were shown in Fig. 9. Abundant expression levels of caspase-3, IL-1, and CHOP were significantly greater in rat treated with *Rhizoma Paridis*, whereas less COX IV and BIP were observed in the treated group as compared to the control group.

## Discussion

Several reports have revealed that unreasonable application of *Rhizoma Paridis* in clinic could cause a risk for development of severe hepatotoxicity. At present, the mouse model has been recognized as a standard animal model, the most intuitive feature of which was the great changes in the tissue structure of liver cells and the rapid increase of both ALT and AST. In the current study, *Rhizoma Paridis* could cause significant elevation of ALT and AST, and a reduction in the increased tendency of body weight and liver weight compared with the control. Previous evidence indicates that fat accumulation mediated the oxidative stress injury, inflammation, and apoptosis that are involved in *Rhizoma Paridis*-induced hepatotoxicity. More importantly, the findings from the present work clearly indicate that *Rhizoma Paridis* treatment caused obvious liver damage (Fig. 1), evidenced by the dose-dependent liver construct change and hepatic cell swelling. Furthermore, the expression level of apoptosis-associated protein (caspase-3) was dramatically increased in *Rhizoma Paridis*-treated groups. As we know, caspase-3 is activated during cell death and liver injury through reduced activity of mitochondria^14^. NOX and SDH, as the key complexes of mitochondrial respiratory chain, were robustly down-regulation in the treated group. Significant elevation in the level of caspase-3 and decreased SDH activity in treated rats  are the evidences of mitochondrial injury induced by *Rhizoma Paridis*.

To explore the biological connotation of alteration proteins, the KEGG and Gene Ontology (GO) databases were conducted to obtain an enrichment analysis result. Interestingly, the most significantly enriched biological process was related to lipid metabolism, degradation, and transport, including the metabolic pathways (rno01100), retinol metabolism (rno00830), insulin resistance (rno04931), adipocytokine signaling pathway (rno04920), PPAR signaling pathway (rno03320), steroid hormone biosynthesis (rno00140), and peroxisome (rno04146). As we know, lipid metabolism is closely related to oxidative stress response in liver injury. Entertainingly, the alteration of glutathione metabolism (rno00840) was also observed, implying that there is a dramatic exacerbation oxidative stress injury in the liver during high dose group in comparison to the control group.

Now there is growing evidence showed that lipid metabolism disturbance is one of the important biological mechanisms of *Rhizoma Paridis*-induced hepatotoxicity. The toxicity process analysis identified that the peroxisomal proliferation-related (rno 04146) proteins disorder elicited lipotoxic.

As an NADH/NADPH- or FADH-dependent oxidoreductase, retinol saturase (RetSat) is an important modulator of liver metabolism and can promote adipogenesis^15,16^. A recent study confirmed that overexpression of RetSat could regulate the levels of reactive aldehyde products of lipid peroxidation^17^. Thus, the increased RetSat in this experiment is indicative of lipid metabolism disorders. Alpha-methylacyl-CoA recemase (Amacr), a key enzyme of bile acid synthesis, can catalyze the racemization of bile acid intermediates during the peroxisomal β-oxidation. Furthermore, a recent study suggested that Amacr played an indispensable role in detoxification of α-methyl-branched fatty acids using the Amacr −/−mice^18^. Apolipoprotein A, a member of high-density lipoprotein (HDL), seemed to act as a mediator in lipoprotein degradation and lipid metabolism and lipid transport from other tissues to the liver^19^. In addition, aldo-keto reductase family 1 member D1 (AKR1D1), can invovle in catalytic reaction regarding bile acid synthesis and steroid hormone metabolism with bile acid intermediate as substrate^20^. EPHX2 is associated with plasma lipid and lipoprotein levels^21^ and participates in arachidonic acid metabolism in adipose tissue^22^. Thus, the robustly change in Amacr, APOA1, AKRID1, and EPHX2 levels in this experiment might be associated with impaired lipid metabolism after exposure to *Rhizoma Paridis*. Notably, increased 3-hydroxybutyrate levels, as observed in the metabolomic data, is indicative of challenged β-oxidation.

Previous studies of lipid profiles have confirmed that its disorder can elicite inflammatory response, oxidative stress and apoptosis, which is triggered by the regulation of relevant signaling transduction pathways.

Inflammation, a rapidly response to pathogens, is triggered to stop further damage and promote tissue repair in acute liver failure, which subsequently elicite an exacerbation anti-inflammatory phenotype. As known to all, high-density lipoprotein (HDL) can restrain pro-inflammatory cytokine in cell-mediated inflammation. For instance, apoa1, a component of HDL, can block T-cell signals from macrophages and lower the expression level of TNF-α and IL-1, which are the standard inflammatory markers. Furthermore, published studies previously have demonstrated that STAT3 is also referred to the inflammatory response through mediating various inflammatory factors including IL-6/STAT3^23–25^. High-mobility group nucleosome-binding protein 1 (HMGN1) has been identified and characterized as one of the nuclear alarmins, which can definitely enter extracellular space as a result of cell death. Thus, in this study, the increased STAT3 and decreased APOA1, ORNT1, and HMGN1 protein levels showed that the inflammatory response was induced in the treatment of *Rhizoma Paridis*. Furthermore, the proteomic result of the inflammatory defense mechanism that protects mouse liver was in good agreement with the upregulated TNF-α and IL-1β in the western blot. Collectively, these proteins are indicative of a change in inflammatory response induced by *Rhizoma Paridis*.

As a factor involved in oxidative stress release pathways^26^, GSTP may play a crucial part in the sensitivity to drug-induced hepatotoxicity^27^. The expression level of GSTA1 and GSTA2 in present study was up-regulated in the *Rhizoma Paridis*-induced group, indicating that these detoxification enzymes play a key role in *Rhizoma Paridis*-induced hepatotoxicity and the oxidative stress levels were enhanced in the *Rhizoma Paridis*-induced group. Glutathione is a vital biological antioxidant and consists of three amino acids: glutamic acid, cysteine, and glycine^28^. The metabolomic results highlighted the impression of *Rhizoma Paridis* on glutathione metabolism and increased glutamic acid was detected. Furthermore, taurine was lowered compared to the control with its protective responses against oxidative stress^29^. As noted above, the hepatotoxicity to *Rhizoma Paridis* results from systemic oxidative stress and its production might be a protection to oxidative stress. On the other hand, the organism with the weak antioxidant capacity failed to defend against stress and then brought on the cell apoptosis.

Accumulated evidence revealed that to keep ER function stability, the unfolded protein response (UPR) is activated, when ER stress occurs. CHOP, acting upon downstream of the UPR, up-regulates inflammatory response and cellular apoptosis as a chaperone of other proteins^30,31^. Besides, BIP is also regarded as a marker for ER stress and triggers the unfolded protein response^32^. Significant increase of CHOP and BIP occurred following exposure to *Rhizoma Paridis*, demonstrating that *Rhizoma Paridis-*induced hepatotoxicity is probably concerned with ER stress. Here, the levels of proteasome subunit beta type-10 (PSB10) and the chaperone proteins, including protein disulfide isomerases A5 (PDIA5), increased, indicating that the protein homeostasis and regular protein translation folding-degradation processes were disturbed, possibly promoted by failure of the hepatocyte repair mechanism and apoptosis^33^.

Notably, mitochondrial dysfunction might be an important clue in understanding the hepatotoxicity induced by *Rhizoma Paridis*. Here we found that, disturbing expression of mitochondrial markers, such as NDUFV3, CLYBL, CPT1, PEPCK-C, have affected the metabolism of energy materials.

NADH dehydrogenase (Ubiquinone) flavoprotein 3 (NDUFV3) was down-regulated in the damaged liver, which plays a crutial part of the mitochondrial membrane respiratory chain NADH dehydrogenase (Complex I)^34^. As we know, ndufv3 can oxidize NADH and reduce FMN by combining with NADH. This reaction participates in TCA cycle and oxidative phosphorylation, which contribute to energy production of mitochondrial matrix. In this experiment, compared with the control, the dysfunctional mitochondrial respiratory chain, caused by the derangement of these enzymes, reduced ATP content that exacerbates mitochondrial damage and cell apoptosis, which was confirmed by the decreased ATP concentration and decreased SDH activity in the treated group. Also, upreuglation of NDUFV3 can accelerate the pace of reactive oxygen species (ROS) production, which might aggravate oxidative stress injury. On the other hand, dose-dependent growth of the malondialdehyde level, decreased SOD and NOX activity lead to the conclusion of oxidative stress injury as well.

Citramalyl-CoA lyase (CLYBL), encoding a malate/β-methylmalonate synthaseis, is observed to distributed in mammalian mitochondria^35^ and can catabolize the citramalyl-CoA to beget the accumulation of pyruvate and acetyl-CoA that supply the TCA cycle with fuel. When CLYBL is absent, accumulation of acetyl-CoA would inhibit both vitamin B12 and methylmalonyl-CoA mutase (MUT), which may have great influence on many processes. For instance, bloking CLYBL can suppress the methionine cycle and TCA cycle with reducing serine, glycine, folate metabolism and inhibiting branched-chain amino acid catabolism^36^.

Located in the outer mitochondrial membrane, carnitineO-palmitoyltransferase1 (CPT1A) acts as a key mediator for transporting fatty acids across mitochondria membrane^37^. Whereby mitochondria uptake long-chain fatty acids, the β-oxidation is tightly subsequent along with the formation of ATP. CPT1A can expedite formation of acylcarnitine via acyl transfer of the long-chain fatty acyl-CoA^38–40^.. Phosphoenolpyruvate carboxykinase (PEPCK), distributing in cytosol and mitochondria, is a key enzyme triggering gluconeogenesis^41^, comprising PEPCK-C and PEPCK-M^42^. The two enzymatically indistinct isozymes are encoded by Pck1 and Pck2 respectively. PEPCK-C signaling promotes gluconeogenesis and hepatic glucose transport, and overlaps with oxidative pathways and many other biosynthetic^43,44^. Thus, markedly increased CPT1 and PEPCK-C in liver may be relevant to the disorders of energy metabolism in mitochondria which was resulted from the impaired assembly of triglycerides into lipoproteins, expediting gluconeogenic program that produces more glucose.

Supporting these findings, many energy-related metabolites, including blood glucose, α-ketoglutarate, fumaric acid, and 3-hydroxybutyrate were affected in present study by metabolomic analysis. It is generally accepted that NAD + takes part in the TCA cycle, including the production of glucose from pyruvate, the oxidative decarboxylation of isocitrate, the oxidation of malate and so on^45^. α-ketoisovaleric acid and fumaric acid are intermediates in TCA cycles, and a decrease in these metabolism levels is indicative of reduced energy metabolism from aerobic respiration, which is consistent with the depletion of NDUFV3 in the proteome results, inevitably producing severe energy shortage in the liver. To replenish energy, a number of amino acids, especially the branched-chain amino acids (isoleucine), were also used as energy sources by the organism, supporting by markedly accumulation of glutamate and glutamine in livers due to increases in protein digestion. Aspartic acid is the synthetic precursor of isoleucine and participates in the ornithine cycle. The up-regulation of these amino acids demonstrates an adaptive mechanism following *Rhizoma Paridis* treatment, which lead to liver dysfunction. In addition, previous studies proved that ornithine, a non-essential amino acid, can regulate mitochondrial metabolic processes and play a defined-role in the urea cycle^46^. Besides, ornithine aminotransferase (OAT) and Ornithine Transporter (ORNT 1) are two major ornithine interacting proteins. As a transmembrane protein, ORNT1 participates in the ornithine uptake by mitochondria, whereas OAT is associated with amino acid metabolism. Here, we found the ornithine and ornithine aminotransferase (OAT) were both down-regulated, suggesting that the ornithine metabolic pathway is inhibited in the treated group,while the amino acid metabolism enzymes, such as glutathione S-transferase α−2 (GSTA2) and asparagine synthetase (ASNS), were disrupted in the liver.

Furthermore, our data indicated epoxygenase P450 pathway was another enriched GO term (rno00982). CYP450s are major chemical-metabolizing enzymes abundant in the liver and have been reported to regulate redox metabolism of numerous endobiotics^47^ and xenobiotics^48^ in connection with the comprehensive detoxification of toxic effects of various toxicants^49–52^. Some P450 cytochrome levels were altered (CYP3A9 was down-regulated), suggesting that the detoxification pathways response to a xenobiotic stimulus was inhibited. In addition, it is reported previously that AKR1D1 genes can induce the disturbance of biochemical function by regulating P450s pathways^53^. In the current study, AKR1D1 was up-regulated, while CYP3A was down-regulated, implying, for the first time, that P450 might act as a mediator in the *Rhizoma Paridis*-induced hepatotoxicity effect.

## Conclusion

To explore the hepatotoxicity underlying mechanism of *Rhizoma Paridis*, we applied integrative omics analyses. First, the levels of histopathological and biochemical index assessment showed that there is a pronounced disturbance of lipid, glucose, and energy metabolism in the livers of recieving *Rhizoma Paridis* rats. The iTRAQ proteomics and metabolomics study found that abnormal mitochondrial function, including the oxidative phosphorylation pathways and the TCA cycle, was the key determinant of hepatotoxicity. Abnormal expression of mitochondrial function could further activate oxidative stress injury and inflammatory response, finally resulting in cell apoptosis. In conclusion, the prominent hepatotoxicity characteristics of *Rhizoma Paridis* were involved in lipid, glucose, and energy metabolism and the most toxic targets induced by *Rhizoma Paridis* were the mitochondria and the endoplasmic reticulum.

## Supplementary information

Supplementary materials.

## Data Availability

The datasets generated during the current study are available in the PRIDE repository, and the project accession number is PXD016625.
