# Supplementary material for: Integrative analysis of proteomic and metabonomics data for identification of pathways related to Rhizoma Paridis-induced hepatotoxicity
Source: Sci Rep. 2020 Apr 16;10:6540. doi: 10.1038/s41598-020-63632-1 (PMC7162872; doi:10.1038/s41598-020-63632-1)
Supplement: Supplementary file 1 — Supplementary materials. [file 41598_2020_63632_MOESM1_ESM.docx]

# Integrative analysis of proteomic and metabonomics data for identification of pathways related to *Rhizoma Paridis*-induced hepatotoxicity

Chongjun Zhao^1,a^, Mingshuang Wang^1,a^, Jianmei Huang^1^, Zhe Jia^1^, Xia Zhao^1^, Erwen Li^1^, Ziying Wei^1^, Ying Dong^1^, Wenxue Liu^1^, Ting Han^1^, Ying Liu^3^, Farong Li^2,*^, Ruichao Lin^1,*^

1 Beijing Key Laboratory for Quality Evaluation of Chinese Materia Medica, School of Chinese Materia Medica, Beijing University of Chinese Medicine, Beijing, P.R., China

2 Key Laboratory of Ministry of Education for Medicinal Resources and Natural Pharmaceutical Chemistry, National Engineering Laboratory for Resource Developing of Endangered Chinese Crude Drugs in Northwest of China, Shanxi Normal University, Xi'an, China

3 Key Laboratory of Tropical Translational Medicine of Ministry of Education, Hainan Key Laboratory for Research and Development of Tropical Herbs, School of Pharmacy, Hainan Medical

a These authors contributed equally to this paper.

* Corresponding authors: E-mail: [735207319@qq.com](mailto:735207319@qq.com) (FL), and [linrch307@sina.com](mailto:linrch307@sina.com) (RL).

# Design of the metabolomic analysis

## Chemicals and Reagents

Methoxyamine HCl, fatty acid methyl ester (C7-C30, FAMEs) standards, pyridine, and anhydrous sodium sulfate were obtained from Sigma-Aldrich (St. Louis, MO, USA). MSTFA (N-Methyl-N(trimethylsilyl)trifluoroacetamide) with 1% (vol/vol) trimethylchlorosilane (MSTFA, with 1% TMCS), methanol (Optima LC-MS), acetonitrile (Optima LC-MS), hexane, dichloromethane, chloroform, and acetone were purchased from Thermo-Fisher Scientific (FairLawn, NJ, USA). Ultrapure water was produced by a Mill-Q Reference system equipped with a LC-MS Pak filter (Millipore, Billerica, MA).

## Sample Preparation

The untargeted metabolomics profiling was performed on XploreMET platform (Metabo-Profile, Shanghai, China). Briefly, the frozen samples that were harvested and stored in an Eppendorf SafeLock microcentrifuge tube, were mixed with 25 mg of pre-chilled zirconium oxide beads and 10 μL of internal standard. Each aliquot of 50 μL of 50% pre-chilled methanol were added for automated homogenization (BB24, Next Advance, Inc., Averill Park, NY, USA). After centrifugation at 14, 000 g and 4 °C for 20 min (Microfuge 20R, Beckman Coulter, Inc., Indianapolis, IN, USA), the supernatant was carefully transferred to an autosampler vial (Agilent Technologies, Foster City, CA, USA). Each aliquot of 175 μL of pre-chilled methanol/chloroform (v/v=3/1) was added to the residue for the second extraction. After centrifugation at 14,000g and 4°C for 20min (Microfuge 20R, Beckman Coulter, Inc., Indianapolis, IN, USA), each 200μL of the supernatant was carefully transferred to an autosampler vial (Agilent Technologies, Foster City, CA, USA). The remaining supernatant from each sample was pooled to make quality control samples. All the samples in autosampler vials were evaporated briefly to remove chloroform using a CentriVap vacuum concentrator (Labconco, Kansas City, MO, USA), and further lyophilized with a FreeZone freeze dryer equipped with a stopping tray dryer (Labconco, Kansas City, MO, USA).

The sample derivatization and injection were performed by a robotic multipurpose sample MPS2 with dual heads (Gerstel, Muehlheim, Germany). Briefly, the dried sample was derivatized with 50 μL of methoxyamine (20 mg/mL in pyridine) at 30°C for 2 hr, followed by the addition of 50 μL of MSTFA (1% TMCS) containing FAMEs as retention indices at 37.5 °C for another 1 hr using the sample preparation head. In parallel, the derivatized samples were injected with sample injection head after derivatization.

## Instrument

A time-of-flight mass spectrometry (GC-TOF/MS) system (Pegasus HT, Leco Corp., St. Joseph, MO, USA) with an Agilent 7890B gas chromatography and a Gerstel multipurpose sample MPS2 with dual heads (Gerstel, Muehlheim, Germany). A Rxi-5 ms capillary column (30m×250μm i.d., 0.25-μm film thickness; Restek corporation, Bellefonte, PA, USA) was used for separation. Helium was used as the carrier gas at a constant flow rate of 1.0 mL/min. The temperature of injection and transfer interface were both set to 270 °C. The source temperature was 220 °C. The measurements were made using electron impact ionization (70 eV) in the full scan mode (m/z 50-500). The detailed instrument settings are briefly described in Table X-2. Instrument optimization was performed every 24 hours.

**Table S1.** The detailed instrument settings of GC-TOF/MS.

| GC | |
| --- | --- |
| Column | Rxi-5MS (crossbond 5% diphenyl/95% dimethyl polysiloxane)  30 m (length) × 250 µm L.D. 0.25-µm film thickness |
| Over programmed temp (℃) | 80 (2 min), 80–300 (12℃/min), 300 (4.5) min, 300-320 (40℃/min), 320 (1 min) |
| Inlet temp (℃) | 270 |
| Injection vol (µl) | 1.0 (splitless) |
| Carrier gas | Helium (99.9999%) |
| Transfer interface temp (℃) | 270 |
| Flow rate (mL/min) | 1.0 |
| Mass spectrometer | |
| Ionization model | Electron impact |
| Electron energy (EV) | -70 |
| Detector voltage (v) | -1450 |
| Source temp (℃) | 220 |
| Acquisition rate | 25 spectra/sec |
| Mass range (Da) | 50–550 |

## Metabolite Annotation

Metabolite annotation was performed by comparing the retention indices and mass spectral data with those previously generated from reference standards of known structures present in JiaLib metabolite database using the proprietary software XploreMET. The current JiaLib comprises over 1,200 mammalian metabolites and is one of the most comprehensive metabolite libraries in the world. The reference chemicals present in JiaLib were commercially purchased from Sigma-Aldrich (St. Louis, MO, USA), Santa Cruz (Dallas, TX, USA), Nu-Chek Prep (Elysian, MN, USA), and synthesized in the laboratory.

## Data Analysis Software

XploreMET (v3.0, Metabo-Profile, Shanghai, China) is a powerful 1-STOP solution for GC-MS-based metabolomics through more than a decade of research and development. The software streamlines procedures for raw data processing, peak deconvulation, compound annotation, statistical analysis, pathway analysis, and project report within minutes of completing the analytical sequence.

## Raw Mass Spectral Data Processing

The raw data generated by GC-TOF/MS were processed using XploreMET for automated baseline denosing and smoothing, peak picking and deconvultion, creating reference database from the pooled QC samples, metabolite signal alignment, missing value correction and imputation, and QC correction.

## Data Preprocessing

Each data set was transformed into comparable data vectors for statistical analysis. All measurements were mean-centered and scaled by the standard deviation of the observed measurements.

**Table S2.** The sequences of the primers

| Gene | Forward primer **(**5’-3**’)** | Reverse primer（5-3） | Accession number |
| --- | --- | --- | --- |
| Ndufv3 | AAgATgTggTgggAgCAg | GAGGAAGAAGAGGCAGCGGTTC | NM_131031 |
| *sdhb* | GGTCACTCGGGCTGGTTT | CTTTCGCCTTGGCTGTTG | NM_130428.1 |
| *Casp8* | CTCGGCGACAGGTTACAG | GGCAGCCAGTTCTTCGTT | NM_022277.1 |
| *Casp3* | CTGGACTGCGGTATTGAG | GGGTGCGGTAGAGTAAGC | NM_012922.2 |
| *Casp9* | CCCCACCCTCACTTTGCT | GGAGGACCAGGCTCACTTA | NM_031632.1 |
| *Bax-2* | GCAAACTGGTGCTCAAGG | GGTCCCGAAGTAGGAAAGG | / |
| *Bcl-2* | CGGGAGAACAGGGTATGA | CAGGCTGGAAGGAGAAGAT | NM_016993.1 |
| *Bid* | *ACCTGCTGGTGTTCGGCTTTC* | *ACCTGCTTGGGCGAGATGC* | NM_022684 |
| *Mcl-1b* | *AAGAGGCTGGGATGGGTT* | *TTGGTGGCTGGAGGTTTT* | NM_021846.2 |
| *ucp2* | *GACAGAAGGATTGCCGAAAC* | *TCATCAAGCCAGCCGAGA* | NM_012682.2 |
| *gstp1* | *CACCTGGGTCGCTCTTTA* | *CTTTGCCTCCCTGGTTCT* | NM_012577.2 |
| *acaca* | TGAAGGGCTACCTCTAATG | TCACAACCCAAGAACCAC | NM_022193.1 |
| *me* | TGGGCATTCTGGGTCAAA | TCCGCTCGTCCACTGTCCTC | M35258.1 |
| *fasn* | *ATCACCCGACTTCCTCTG* | *TGAATACGACCACGCACTA* | NM_017332.1 |
| *g6pd* | TTGGCAGCGGCAACTAAA | GCGGGCATAGCCTACAAT | NM_017006.2 |
| *acadm* | *GGAGCACCAAGGAGTTTC* | *AATCCATAGCCTCCGAAA* | NM_016986.2 |
| *acox1* | *GGGCACGGCTATTCTCAC* | *GACTCGGCAGGTCATTCA* | NM_017340.2 |
| *cpt1* | TGGGCTTACTTCTTCATTC | AGGTTTGTTTGGCTGTTT | AF020776.2 |
| *mfn2* | TAGGGTGTCCGATGTGGT | TAGCAAGGCAGGGATGAG | NM_130894.4 |
| *lamp2* | :CCATTGGGTGTCATCTTT | AGTTGGAACGGGTATTGA | NM_017068.2 |
| *lc3a* | GTCTATGCCTCCCAAGAAACC | CCCAGCCAGCACCCAAAA | NM_199500.2 |
| *parkin* | ACGCTCAACTTGGCTACTC | CACTCCTCGGCACCATAC | AB039878.1 |
| *pink* | ACCTATGCCCATCCATCTAA | AGTGTCATCCGTGTTTGTCC | NM_001106694.1 |
| *nix* | TCCTCGTCTTCCATCCAC | TCTGCCCATCTTCTTGTG | NM_080888.1 |
| *pdha1* | *GCGTTTCTCATCCGTTGT* | *ATGGCAGAGTTGTTCCTTTT* | NM_001004072.2 |
| *IL-1β* | GGTGGTGGTTCTCATCGT | TGTCAGCAGCACAAACTCT | NM_031512.2 |
| *TNF-α* | CCACGCTCTTCTGTCTACTG | GCTACGGGCTTGTCACTC | NM_012675.3 |
| *Atf6* | GCAGGTGTATTACGCTTCG | TTCGGTCTTGTGGTCTTGTT | NM_001107196.1 |
| *ern1* | ATGTGAGTGACCGAATAGAAA | CAGCGTCTCCTGAACCTC | NM_001191926.1 |
| *eif2ak3* | TGTCTTGGTTGGGTCTGA | TTCTTGCGGATGTTCTTG | NM_031599.2 |
| *eif2s1* | AAGGGAGTGGCTTGATTT | AGGTTGGGATTGTTGGTG | NM_019356.1 |
| *hsp90* | TACTCGGCTTTCTCGTCA | AGGGCATCAGAAGCATTA | AY695393 |


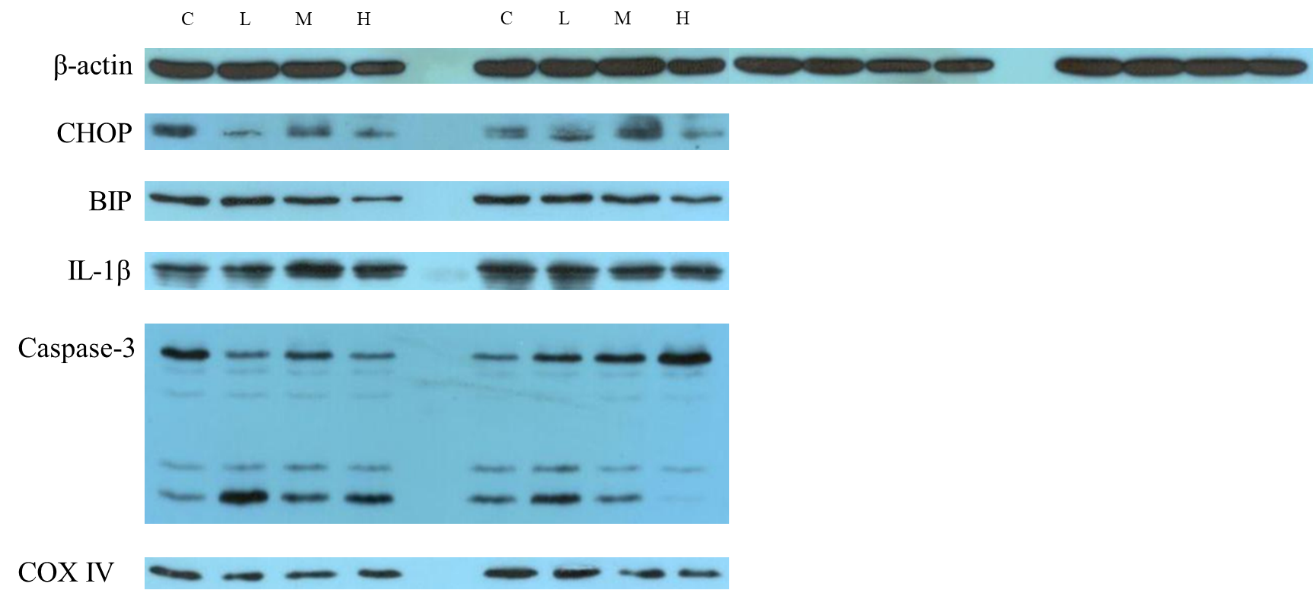


Figure S1. Full-length blots of CHOP, BIP, IL-1β, Caspase-3 COX IV concentrations in liver tissue from control and treated rats.
